# Conceptual evolution of hypospadiology

**DOI:** 10.4103/0970-1591.40613

**Published:** 2008

**Authors:** Amilal Bhat

**Affiliations:** Department of Urology, S.P. Medical College, Bikaner, Rajasthan 334003, India. E-mail: amilalbhat@rediffmail.com

Surgical correction of hypospadias is an ultimate challenge in urethral reconstruction. The discipline of hypospadias surgery requires passion and single-minded dedication apart from surgical dexterity and in-depth knowledge of different surgical techniques. Professor Asopa from Agra has contributed immensely to the development of hypospadias surgery and has been instrumental in encouraging many to take up this challenge and I remain indebted to him for his constant encouragement and guidance.

In the 1970s and 1980s when single-stage flap procedures became popular the general consensus was that the problems of hypospadias repair had been solved. The same euphoria was again felt when Snodgrass popularized tubularized incised plate (TIP), but the rebirth of two-stage repair has taken us back to square one. This shows that the hypospadiology is still in evolution stage. The complexity in management and variability in reproducibility of results in different hands limit urologists to take up this surgery in their surgical armamentarium.

There have been many conceptual changes in hypospadias management, starting from amputation of penis distal to meatus, to replacement of urethra by tissue culture techniques. In the era of two-stage repair, the goal in management used to be reconstruction of neourethra and chordee correction. Complete correction of chordee and location of meatus at the tip of the glans were not considered necessary and minimal chordee with coronal meatus was acceptable. Improved results of hypospadias repair in the last three decades has led us to think about the shape of the glans and shaft, slit like meatus at the tip and impact of repair on psychological, sexual, and fertility problems. More emphasis is being laid on both cosmesis and functional aspects of reconstruction. Even minimal residual curvature and torsion are not acceptable. Recently role of preputioplasty in hypospadias repair is increasing as circumcised penis is not acceptable to many. With more than 300 procedures and variable results, the beginner of hypospadias surgery is in a dilemma for the best suitable procedure. This symposium on hypospadias is the need of the times and would aim to provide with practical guide lines in management. I am happy that stalwarts in this field have agreed to contribute toward this symposium and I am indeed indebted to all of them.

Nowadays more emphasis is laid on glanuloplasty and meatoplasty which produce a conical glans with the meatus at the tip and symmetry with the penile shaft and additionally reduce the incidences of fistula, diverticula, and meatal stenosis. Extended application of TIP in proximal hypospadias and redo cases has enhanced results in the form of cosmesis as well as good function. The word chordee is synonymous to fibrosis, but it has now been proved beyond doubt that the abnormal tissue found is spread out corpous spongiosum, rather than fibrous tissue. So the new terminology for bending deformity of penis is “Penile Curvature.” These facts are highlighted by Snodgrass in his article.

A very important evolution has been in improvement of pediatric anesthesia that allows us to operate upon the child at a younger age, giving the advantages of better healing and sparing the child of carrying the mental trauma of surgery to adulthood.

Advances in fine suture materials, microsurgical instruments, and use of magnification have improved the results of hypospadias surgery. Another important conceptual change, which has had a positive impact on outcome of surgery, has been to cover the neourethra with healthy tissue. Various tissues used are dartos (dorsal, ventral, or adjacent) denuded skin, tunica flaps, and spongiosum. Spongioplasty has been added to reduce the complications and improve cosmesis, and has gained popularity. These issues are discussed in general consideration in hypospadias repair by the author.

The choice of procedure in distal hypospadias repair is TIP as shown in the article of Snodgrass and Braga. But controversy still continues in management proximal hypospadias. One school of thought prefers single-stage repair while other rely on two-stage repair. I hope articles of Snodgrass, Mouriquand, Jayanthi, Bracka, and Manzoni will give reader the guidelines to choose the right procedure in a given case.

Correction of curvature is an important step in single-stage repair. The controversy whether to shorten the dorsal surface or to lengthen the ventral is likely to be solved by step-by-step approach given by the author.

The complication rate in hypospadias repair varies from 0 to 50%. Many of these could be the sequelae of acute postoperative complications. The author has elaborated the incidence, causes, and methods for preventions with an audit to reduce the complications. The issue of infertility and sexual dysfunction after hypospadias repair is well taken up in the article of Dr. Chandra Singh. Chad Wallis has compared the use of grafts and flaps in hypospadias repair with their outcome.

In future “Robotics” in hypospadias surgery may prove to be an important milestone, by eliminating human tremor factors and effecting accurate approximation of tissues with minimal trauma, thereby reducing complications and improving results.

I hope these articles will illuminate all important aspects of hypospadias and help readers to gain a better understanding of this complex subject and leave them more confident in tackling the problems of hypospadias reconstruction.

I am confident that this symposium will help encourage more of our colleagues to take up hypospadias surgery earnestly in their practice. Last but not the least, I would like to express my sincere gratitude to the Editor of *Indian Journal Urology* for the invitation to guest edit this symposium and I sincerely thank him for the same.

